# Next-generation sequencing yields the complete chloroplast genome of *Pleione forrestii* (Orchidaceae)

**DOI:** 10.1080/23802359.2019.1637288

**Published:** 2019-07-31

**Authors:** Sha-Sha Wu, Xiao-Qian Wu, Na Dong, Rui Ling, Hong Jiang

**Affiliations:** a Key Laboratory of National Forestry and Grassland Administration for Orchid Conservation and Utilization at College of Landscape Architecture, Fujian Agriculture and Forestry University, Fuzhou, China;; b Fujian Ornamental Plant Germplasm Resources Innovation & Engineering Application Research Center, Fuzhou, China;; c Yunnan Academy of Forestry, Kunming, China

**Keywords:** Chloroplast genome, phylogenetic, Illumina sequencing, *Pleione forrestii*

## Abstract

*Pleione forrestii* is an endangered terrestrial orchid with significant ornamental values. Here, we report the first complete chloroplast genome of *P. forrestii*. The circular genome was 158,759 bp in length and consisted of a pair of inverted repeats (IR 26,429 bp), which were separated by a large single-copy region (LSC 87,236 bp) and a small single copy-region (SSC 18,665 bp). It contained 135 genes (115 unique), including 87 protein-coding genes, 38 tRNAs, and 8 rRNAs. The maximum likelihood phylogenetic analysis indicated that *P. forrestii* was sister to *P. bulbocodioides* and *P. formosana*.

*Pleione forrestii* is a lithophytic or an epiphytic herb, growing on humus-covered rocks and tree trunks in open forests and at forest margins, distributed between 2200 and 3200 m in northern Burma and northwestern Yunnan (Cribb and Butterfield 1999; Chen et al. 2009). Because of somewhat restricted in its distribution and over collection (Cribb and Butterfield 1999), this species has been listed as an endangered species in the Red List (IUCN 2018). Moreover, it is the only yellow-flowered species and an important parent in cross-breeding, which has been used to introduce yellows into a succession of hybrids. Thus, it will be fundamental to formulate conservation and management strategies for this threatened species. In this study, we assembled and characterized the complete plastome of *P. forrestii*.

Total genomic DNA was extracted from fresh leaves (26°0′49.90″N, 98°37′22.79″E, LSForrest 01, FAFU) using a modified CTAB method (Doyle and Doyle 1987) and sequenced by the Illumina Hiseq 2000 sequencing platform. The specimen’s DNA was stored in Fujian Agriculture and Forestry University. Raw reads were filtered using NGS QC Toolkit (Patel and Jain 2012). The clean reads were first aligned to *P. bulbocodioides* (Genbank Accession No. KY849819) (Shi et al. 2017). Filtered reads were then assembled into contigs in the software CLC Genomics Workbench v8.0 (CLC Bio, Aarhus, Denmark). After assembled, the obtained scaffolds and contigs were assembled into cp genome by Geneious 11.1.15 (Kearse et al. 2012) using the algorithm MUMmer. The genome was automatically annotated using DOGMA (Wyman et al. 2004), then adjusted by Geneious version 11.1.15 (Kearse et al. 2012) and submitted to GenBank with the accession number MK370035. The cp genome sequence of *P. forrestii* is 158,759 bp in length, containing a large single-copy (LSC) region of 87,236 bp and a small single-copy (SSC) region of 18,665 bp, and two inverted repeat (IR) regions of 26,429 bp. The cp genome encoded 135 genes, of which 115 were unique genes (87 protein-coding genes, 38 tRNAs, and 8 rRNAs). Overall GC content of the whole genome is 37.2%, while the corresponding values of the LSC, SSC, and IR regions are 35.0, 30.3, and 43.3%, respectively.

To further investigate its phylogenetic position, 85 complete cp genomes of Epidendroideae and two species of Orchidoideae were aligned using HomBlocks pipeline (Bi et al. 2017). RAxML-HPC Black-Box version 8.1.24 (Stamatakis et al. 2008) was used to construct a maximum likelihood tree with *Ludisia discolor* and *Goodyera fumata* as outgroup. The branch support was computed with 1000 bootstrap replicates. The ML tree analysis indicated that *P. forrestii* was sister to *P. bulbocodioides* (KY849819) and *P. formosan*a (MK361027) with 100% bootstrap support (Figure 1).

**Figure 1.**
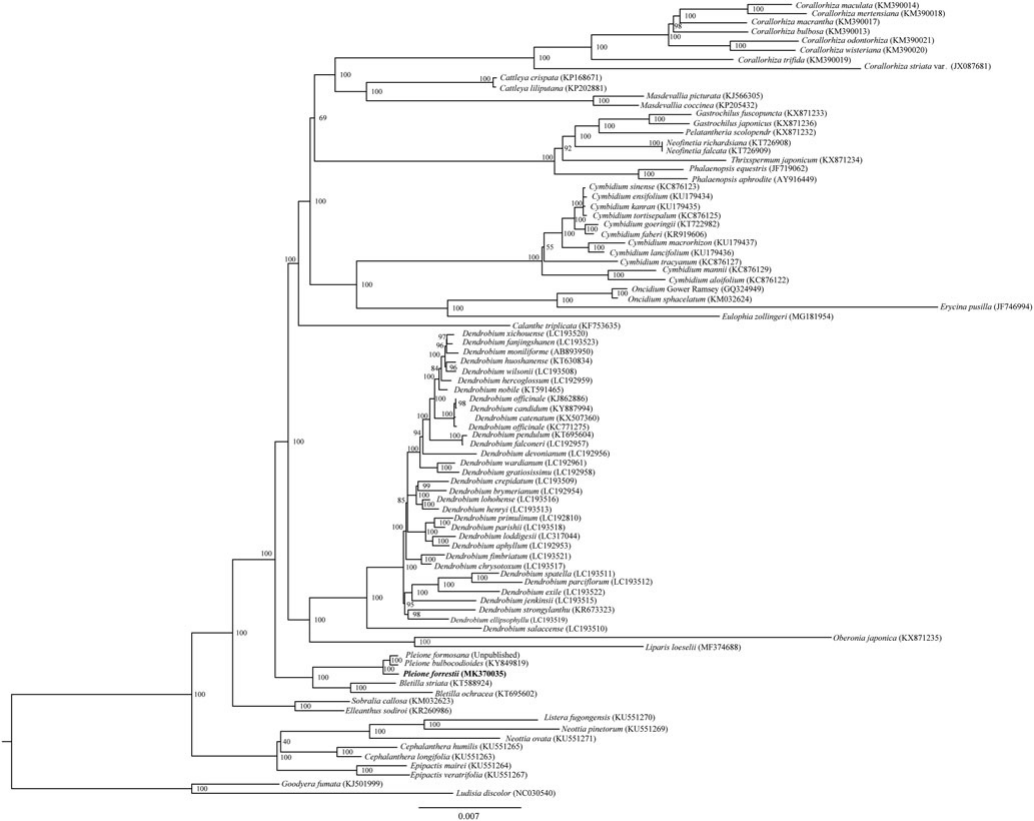
The maximum-likelihood (ML) tree based on 85 species within Epidendroideae, based on whole chloroplast genome sequences, with *Goodyera fumata* and *Ludisia discolor* (Orchidoideae) as outgroup. The bootstrap value based on 1000 replicates is shown on each node, and the position of *Pleione forrestii* is shown in bold.
